# Quality of life in people with dementia living in nursing homes: validation of an eight-item version of the QUALIDEM for intensive longitudinal assessment

**DOI:** 10.1007/s11136-020-02418-4

**Published:** 2020-01-18

**Authors:** Stefan Junge, Paul Gellert, Julie Lorraine O’Sullivan, Sebastian Möller, Jan-Niklas Voigt-Antons, Adelheid Kuhlmey, Johanna Nordheim

**Affiliations:** 1 Institute of Medical Sociology and Rehabilitation Science, Charité – Universitätsmedizin, Charitéplatz 1, 10117 Berlin, Germany; 2 Quality and Usability Lab, Technical University of Berlin, Ernst-Reuter-Platz 7, 10587 Berlin, Germany

**Keywords:** Caregiving, Well-being, Nursing home, Quality of life, Alzheimer’s disease, Touchscreen tablet

## Abstract

**Purpose:**

Our aim was to examine whether quality of life which was repeatedly assessed over time is related with the comprehensive assessment of quality of life (QoL) and thereby to validate a brief QoL assessment.

**Method:**

This longitudinal study used a comprehensive assessment of quality of life at baseline (QUALIDEM; 37 items) to validate an eight-item version of QUALIDEM to assess momentary quality of life which was repeatedly administered using a tablet device after baseline. In all, 150 people with dementia from 10 long-term facilities participated. Momentary quality of life and comprehensive quality of life, age, gender, activities of daily living (Barthel Index), Functional assessment staging (FAST), and Geriatric Depression (GDS) have been assessed.

**Results:**

Comprehensive and momentary quality of life showed good internal consistency with Cronbach’s alpha of .86 and .88 to .93, respectively. For multiple associations of momentary quality of life with the comprehensive quality of life, momentary quality of life was significantly related to comprehensive quality of life (*B* = .14, CI .08/.20) and GDS (*B* = − .13, CI − .19/− .06). More specifically, the comprehensive QUALIDEM subscales ‘positive affect’, ‘negative affect’, ‘restlessness’, and ‘social relationships’ showed significant positive associations with momentary quality of life (*p* < .001).

**Conclusion:**

We found that momentary quality of life, reliably assessed by tablet, was associated with comprehensive measures of quality of life and depressive symptoms in people with dementia. Broader use of tablet-based assessments within frequent QoL measurements may enhance time management of nursing staff and may improve the care quality and communication between staff and people with dementia.

**Electronic supplementary material:**

The online version of this article (10.1007/s11136-020-02418-4) contains supplementary material, which is available to authorized users.

## Introduction

As there is not yet a curative treatment for dementia, a major goal of caring for people with dementia (PwD) is the maintenance of quality of life (QoL). In the USA [1, 2] as well as in Germany [3], about half of the older adults aged 65 and more living in nursing homes are diagnosed with dementia, which is 19-fold higher than the prevalence of dementia in individuals over 65 living in the community [3].

Even though there is a consensus about the importance of QoL as a goal of care in PwD, there is still a debate about theory, assessment, and factors associated with QoL in PwD [4]. However, so far only a few assessment tools are based on theory; most were proxy rating compared to self-rating and conceptualized QoL as general health related or domain specific [5]. Therefore, reliable instruments to assess QoL are necessary. In a recent systematic review of QoL in PwD, three factors that are positively associated with a better QoL were having close relationships, social engagement, and functional abilities. [6]. Focusing on the psychosocial domains of QoL in PwD, the QoL assessment tool QUALIDEM showed the best acceptability interviewing PwD and their proxies. It was considered as acceptable in the long and short forms profiling PwD with mild-to-very severe stages of dementia living in nursing homes [4, 7–9].

Although many established QoL instruments allow a comprehensive assessment of QoL in PwD, momentary variations in QoL across time may be uncaptured [10]. Ecological Momentary Assessment (EMA) showed accuracy, minimization of retrospective bias, and revealing dynamic processes as compared to more traditional comprehensive QoL assessments across studies [11, 12]. Having a positive mood and being in social interaction assessed on a momentary level, i.e., assessed on a daily basis, has been related to a higher level of QoL. These findings were assessed on a comprehensive-level QoL (i.e., by the QoL-AD) in the Maastricht Electronic Daily Life Observation (MEDLO) study [13] as well as in other intervention studies in PwD [14]. These studies provided first hints about the association of momentary and comprehensive assessment of QoL.

New technologies may help to assess QoL in PwD due to the frequent QoL measurements across time. Previous studies suggest the feasible use of technology-based and more specifically touchscreen-based assessments for elderly people, PwD, and other people with neurodegenerative disorders [15–17]. Other studies investigated the use of smartphones to measure the momentary QoL in PwD and people with cognitive impairment and identified a good acceptability, feasibility, and accuracy as well [18–21]. However, further research is needed that examines the relation of momentary QoL with comprehensively assessed QoL in PwD.

## Aims of the study

The aim of our study was to validate a brief version of the QUALIDEM that would be suitable for momentary assessment by analyzing the association of momentary (assessed at several time points) and comprehensive QoL in PwD living in nursing homes. We investigated factors that were associated in the eight-item and the 37-item version of QUALIDEM at baseline measurements. Inspecting correlations of those two scales may help us to enhance our knowledge on the mechanisms of QoL over time and may be helpful for the nursing staff to assess QoL in the future. In the first step, we aimed at testing the momentary and time-lagged reliability of a momentary assessment of QoL. Furthermore, we hypothesized a positive association of momentary QoL with comprehensive QoL. Additionally, we hypothesized that the relationship between momentary and comprehensive QoL exists when adjusting for age, gender, cognitive status, functional status, and depressive symptoms as well as temporal trend and between-facility variation.

## Methods

### Study design

The PflegeTab (*engl.* CareTab) study aimed to develop and evaluate a tablet-based psychosocial intervention tailored to the needs of PwD. A tablet application was developed and combined with innovative care concepts in order to enable a flexible, patient-centered care approach. The app was tested in ten nursing homes in Berlin over an 8-week intervention period, in which participating residents received activation sessions 3 times per week for up to 30 min. The full 37-item version of the QUALIDEM questionnaire was used for the assessment of comprehensive QoL before and after the intervention period. In the present analyses in this article, we only regarded the individual measurements that were administered before the intervention (at baseline). As our main focus lay on momentary and comprehensive QoL, momentary QoL was measured via tablet during the intervention period before and after each activation session (For the present analyses, before session assessments were used only; Table 1).

**Table 1:** Sample characteristics

Scale	Mean (SD)	Empirical range	Items	*N*	Cronbach’s *α*
Age, years	84.9 (7.1)	53–100	1	150	–
Women, %	75		1	112	–
Depressive symptoms, GDS	3.9 (2.9)	0–15	15	111	.68
Functional status, Barthel Index/ADL	54.1 (26.3)	0–95	10	149	.89
Dementia stage, FAST	9.0 (1.9)	4–16	16	149	.75
Baseline momentary QoL	5.4 (1.2)	1.6–7.0	8	150	.89
Baseline comprehensive QoL (sum score)	83.3 (14.9)	43–115	37	149	.86
*QUALIDEM subscales*					
Care relationship	15.5 (4.6)	1–21	7	148	.84
Positive affect	13.1 (3.7)	1–18	6	148	.86
Negative affect	6.4 (2.2)	0–9	3	148	.77
Restlessness	5.6 (2.6)	0–9	3	149	.63
Positive self-image	6.9 (2.0)	1–9	3	148	.49
Social relationship	11.5 (3.8)	1–18	6	149	.74
Social isolation	6.8 (1.9)	1–9	3	149	.42
Feeling at home	9.1 (2.6)	2–12	4	143	.60
Having something to do	2.2 (1.6)	0–6	2	149	.20

*SD* standard deviation, *GDS* Geriatric Depression Scale; *FAST* functional assessment staging; *ADL* activity of daily living; *QoL* quality of life

### Participants and procedure

**Fig. 1:**
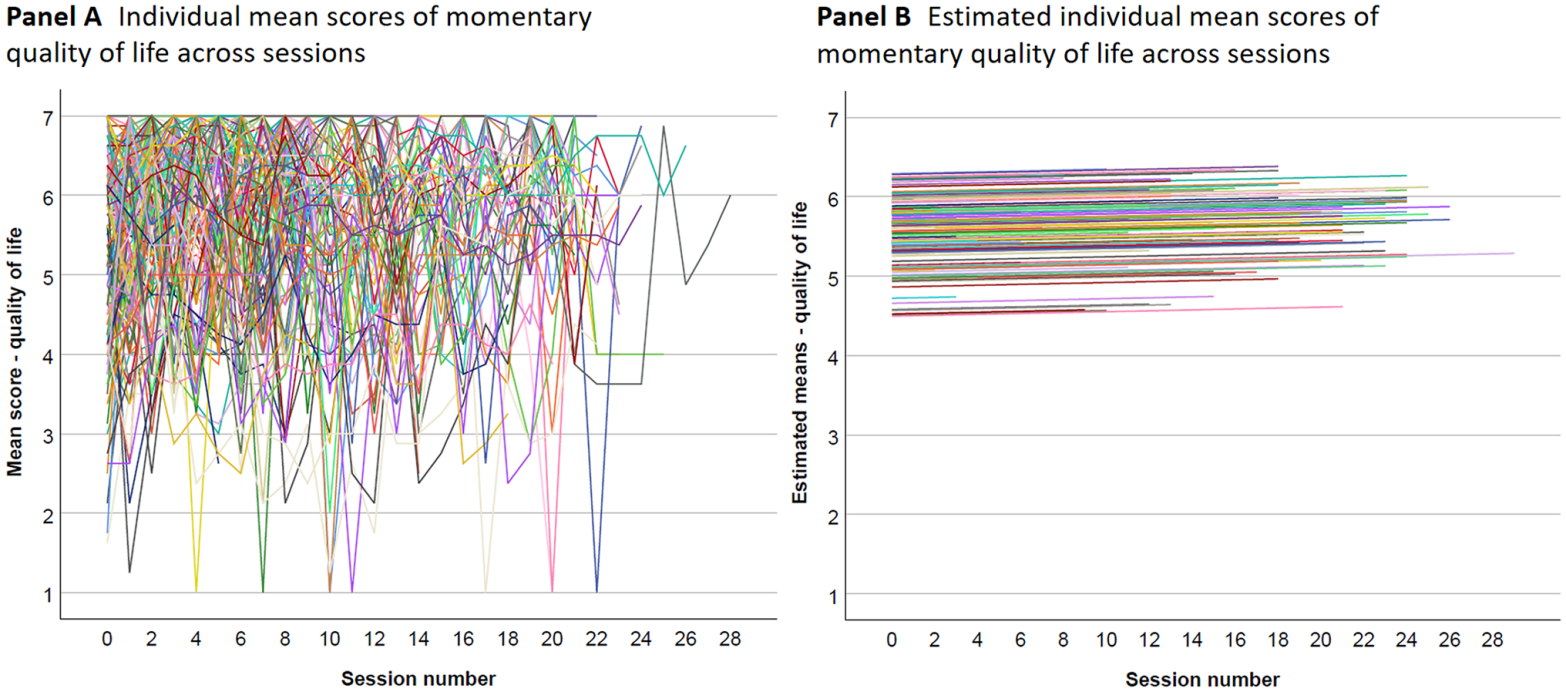
**a** Displays the individual mean scores of momentary quality of life across sessions. **b** Shows the predicted individual mean scores of momentary quality of life across sessions estimated by a multivariate model with fixed effect (linear) of session number, random intercept, and slope

The planned sample size of the PflegeTab study was *N* = 240 PwD across eight nursing homes [22]. The sample size calculation was based on the main study, which included a randomized trial design, where the sample size referred to a medium-large effect size of Cohen’s *d* = 0.5 (*α* = 0.05; 1 − *β* = 0.80; 20% dropout rate; between cluster variance ICC = 0.005; G*Power 3.1) between the two arms where we primarily used the assessments at baseline (see ISRCTN98947160). Although the number of participating care facilities was increased to ten during the planning phase, the target sample size could not be fully achieved. Participants were included if they were nursing home residents and had a medical diagnosis of dementia (International Statistical Classification of Diseases and Related Health Problems—ICD-10: F00-F03), including Alzheimer’s disease, vascular dementia, and unspecified dementia [23]. Participants were excluded if other serious chronic psychiatric diagnosis were given F10-29, exceptions: F10.1; F17.1; F17.2; F32.2; and F32.3 may be included. An admission to the nursing home less than 4 weeks beforehand was also a criterion for exclusion. A total of 203 people (eligible nursing home residents or their conservators) were contacted and 163 PwD took part in the PflegeTab study. For the current analyses, 13 of the participants could not be included because the baseline momentary QoL and momentary QoL could not be imputed. As QUALIDEM is a proxy-rated assessment tool, all participants were assessed by the nursing staff working in the nursing homes (Fig. 1).

### Measures

*Comprehensive QoL* was assessed with a 37-item version of QUALIDEM suitable for PwD with mild-to-severe dementia using proxy rating [24]. Out of 40 existing items, Item 9: ‘Does not want to eat’, item 30: ‘Likes to lie down in bed’, and item 15: ‘Enjoys meals’ was not represented because we excluded the subscale J in our study; thus, 37 items remained. In order to test the main hypothesis, a sum score was created across all 37 items at baseline. Further, we studied the determinants of QoL according to the literature [25–27] to establish nine subscales: care relationship (7 items), positive affect (6 items), negative affect (3 items), restlessness (3 items), positive self-image (3 items), social relationship (6 items), social isolation (3 items), feeling at home (4 items), and having something to do (2 items) (Table). These subscales are sum scores across the respective items, which were presented in a Likert format ranging from never (0), rarely (1), and sometimes (2) to frequently (3). Across all 37 items, a principal component analyses (PCA) extracted ten components with an Eigenvalue over 1 although the visual evaluation of the Scree plot suggested four components only. The varimax-rotated PCA solution accounted for 67.2% of the overall variance. Next to the subscales, we created a composite sum score across all 37 items, which has been used extensively in the literature as well [25–30]. Thus, in our analyses, we look into both the comprehensive sum score (Table 2) as well as subscale results (Table 3) and its relation with momentary QoL.

**Table 2:** Univariate and multiple associations with momentary quality of life

	Univariate model				Multiple model			
	*B*	95% CI		*P-*value	*B*	95% CI		*P*-value
Comprehensive QoL	**.17**	**.11**	**.23**	** < .001**	**.14**	**.08**	**.20**	** < .001**
Session, linear	.05	− .01	.11	.123	.03	− .02	.09	.264
Age	− .06	− .12	.01	.081	− .06	− .13	.00	.054
Gender, women	.01	− .05	.07	.723	.03	− .03	.09	.321
Barthel Index	− .006	− .07	.06	.859	− .02	− .11	.06	.602
GDS score	− .**16**	− .**22**	− .**10**	** < .001**	− .**13**	− .**19**	− .**06**	** < .001**
FAST score	.04	− .03	.10	.240	.04	− .04	.13	.358
*Random effects*								
AR diagonal	**.90**	**.84**	**.96**	** < .001**	**.88**	**.82**	**.95**	** < .001**
AR rho	**.39**	**.35**	**.43**	** < .001**	**.37**	**.33**	**.41**	** < .001**
Variance between facilities	.08	.03	.22	.062	.09	.03	.25	.058

*B* z-standardized B coefficient that can be interpreted as standardized beta coefficient, *95% CI* lower and upper limit 95% confidence interval, *P-*value level of significance (Significant values are shown in bold), *AR diagonal* random variance of session of measurements between individuals, *AR rho* residual correlation between sessions of measurement, *QoL* quality of life, *GDS* Geriatric Depression Scale, *FAST* Functional assessment staging. *N* = 148 across models (two cases could not be imputed)

**Table 3:** Multiple associations of momentary quality of life with comprehensive QUALIDEM subscales

QUALIDEM subscale	*B*	95%CI		*P*-value
Care relationship	.02	− .04	.08	.535
Positive affect	**.17**	**.11**	**.23**	** < .001**
Negative affect	**.13**	**.07**	**.19**	** < .001**
Restlessness	**.07**	**.01**	**.14**	**.023**
Positive self-image	− .01	− .07	.05	.836
Social relationships	**.16**	**.09**	**.22**	** < .001**
Social isolation	**.07**	**.01**	**.14**	**.027**
Feeling at home	.04	− .02	.11	.212
Having something to do	.03	− .04	.09	.441

*B* z-standardized B coefficient that can be interpreted as standardized beta coefficient.* P-*value level of significance (Significant values are shown in bold). Coefficients of each subscale were derived from a separate model, which have been adjusted for age, gender, time, GDS, Barthel Index, and FAST as described in the analysis section. *N* = 148 across models (two cases could not be imputed)

*Momentary QoL* was assessed with an eight-item version from the 37-item long version of QUALIDEM. All eight items had been executed on tablet computers at repeated time points. Aiming to use a brief scale that was considered as suitable for the tablet-based momentary QoL assessment in real life nursing home environment by nurses, we chose eight items: restlessness [item 19 of the QUALIDEM], mood [item 10], anxiousness [item 6], body language [item 22], communication [item 12], happiness [item 05], sadness [item 11] and sociability [item 34] belonging to the four subscales: ‘positive affect’, ‘negative affect’, ‘restlessness’, and ‘social relationships’ of the full 37-item version of QUALIDEM (i.e., subscale B, subscale C, subscale D, and subscale F) [8, 31]. As part of a workshop, the study team was selecting the items based on the following considerations: Two items from four subscales of the German QUALIDEM version were used for the short version. In addition, some scales were not suitable for nursing home residents with severe stages of dementia. The item length played a minor role and was relevant only when selecting the two items from a scale. In that case, shorter items were preferred, since we assumed that all items belonging to the same scale were assumed equivalent in content and therefore largely interchangeable.

*Dementia stage* was measured with the Functional assessment staging (FAST) which is scored from 1 to 16 for 7 consecutive stages and 9 substages [32]. As PwD progress in severity, the numerical value of the FAST increased, with stage 4 corresponding to mild dementia, stage 5 to moderate, stage 6 to moderately severe, and stage 7 to severe dementia. According to Arons et al. (2017), the QUALIDEM is applicable in PwD with all stages of dementia severity [7].

*Functional status* was measured with the Barthel Index [33]. Barthel Index is scored from 0 to 100, served as a covariate and activities of daily living (ADL) related to self-care activities such as bathing, dressing, grooming, and other activities. Barthel Index ranged from 0 to 95.

*Depressive symptoms* were measured with GDS-SF-15 (Geriatric Depression Scale– 15 items Short Form which served as a covariate [34]. Total score ranged from 0–15. A score of 5 or more indicated probable depression. GDS ranged from 0 to 15 points.

*Session (time trend)* refers to the measurement session where the eight-item QUALIDEM was administered. Session variable started from 0 (at baseline; first session, 1, 2, 3,…). Mostly, there were three sessions a week and the median total number of sessions per resident was 8 sessions. Using the session variable, the timing of the momentary assessment can be modeled.

Additional covariates were *ID* (i.e., unique identifier of facility), *age*, and *gender* of the residents.

### Statistical analysis

The univariate and multivariate associations of momentary and comprehensive QoL were estimated using mixed modeling which takes the nested data structure of measurement occasions in individuals in nursing homes into account. Momentary QoL (level-1) was regressed on momentary session (time trend; level-1) and comprehensive variables, i.e., comprehensive QoL, age, gender, functional and cognitive status, and depressive symptoms (all level-2). The ID of the nursing home of each person with dementia was used as a clustering variable (level-3). Intercepts were allowed to vary across individuals and facilities (intercept only model; variance components). Furthermore, an autoregressive covariance matrix was assumed for the time variables to account for autocorrelation (based on inspection of fit indices [AIC; Akaike's Information Criterion [35], where smaller values indicate better fit: AIC_autoregressive_ = 6272; AIC_unstructured_ = 6544, without reaching convergence; AIC_identity_ = 6617; AIC_variance components_ = 6622] and theoretical plausibility). Prior to the estimation of the multivariate models, we inspected the trends graphically as well as compared the AIC values. Based on the inspection, we decided for a fixed slope random intercept model (AIC = 6272) as quadratic and cubic fixed and random effects did not provide improvement in the fit of the model with the data (AIC = 6278 to 6307). All variables have been standardized allowing the coefficients to vary between − 1 and 1 and, thus, can be interpreted as beta coefficients. All data analyses were conducted with MIXED procedures in SPSS (IBM SPSS Statistics for Windows, Version 25.0, released 2017. Armonk, NY: IBM Corp.) using the Restricted Maximum Likelihood estimator (REML), which accommodates for unbalanced datasets, i.e., missing cases in repeated measure designs [35]. Scale missing values were imputed using Expectation–Maximization algorithm for Barthel Index (*n* = 1), FAST (*n* = 1), GDS (*n* = 38), and comprehensive QoL (*n* = 1). Models with and without imputation did not differ in the direction or magnitude of the main findings.

For the evaluation of the internal consistency, at each session (time point), Cronbach's alpha was used (Kuder-Richardson Reliability Coefficients KR20 in the case of binary items). Internal consistency was considered as excellent if Cronbach’s alpha was *α* ≥ .90 or higher, as good if *α* ≥ .80, as acceptable if *α* ≥ .70 and as questionable to unacceptable if *α* ≥ .60 [36]. For test–retest reliability, lagged momentary QoL variables (lag 1, lag 2) were calculated and regressed on momentary QoL in a model that accounted for the nested structure as described above (except for using a variance components covariance structure to capture autocorrelation merely within the coefficients). According to Dichter et al. (2011), test–retest reliability can be seen as the response stability over time, which was operationalized as the regression coefficient of the same test across subsequent time points [37, 38]. Thereby, lag 1 refers to the regression of the value of momentary QoL in one session onto the value of momentary QoL in the prior session. Accordingly, lag 2 refers to the regression of the value of momentary QoL in one session onto the value assessed two sessions before. In the tested models, momentary QoL was compared with each lagged momentary QoL for the nested data structure (Supplementary Table S2).

## Results

The sample compromised of 150 PwD from 10 long-term facilities with a mean age of 84.9 (SD 7.1) years (Table 1). The majority (75%; *n* = 112) were women. Mean GDS score was 3.9 (SD 2.9) and the mean Barthel Index score was 54.1 (SD 26.3). Concerning the dementia stage, the mean FAST score was 9.0 (SD = 1.9) at baseline, which indicates mild cognitive impairment. The mean Comprehensive QoL was 83.3 (SD 14.9), whereas the mean momentary QoL at baseline was 5.4 (SD 1.2). Ranging from 1 to 29 measurement sessions the mean number of sessions (time trend) was 8.6 (SD 6.3).

### Internal consistency

We found that the internal consistency of the comprehensive sum scale of the QUALIDEM was good with a Cronbach’s alpha of *α* = .86. Likewise, the internal consistency of the eight-item momentary QoL was excellent as indicated by Cronbach's alpha with a range from *α* = .88 to *α* = .93, indicating decent reliability at each time point (Supplementary Table S1). Regarding unidimensionality of the momentary QoL scale, a PCA extracted one factor with an Eigenvalue over 1 and the evaluation of the Scree plot suggested one factor as well. The PCA solution accounted for 62.6% (sums of squared loadings) of the overall variance. Regarding test–retest reliability, momentary QoL was associated with each lagged momentary QoL (i.e., lag 1) with *B* = .40 (CI .36/.44, *p* < .001) when accounting for the nested data structure (Supplementary Table S2). Additionally, the lagged association of momentary QoL lagged across two sessions of assessment (i.e., lag 2) was *B* = .36 (CI .31/.40, *p* < .001). Finally, in a model where both (consecutive lag 1 and lag 2 time points momentary QoL) were entered, lag 1 association was *B* = .32 (CI .27/.36, *p* < .001) and lag 2 was *B* = .23 (CI .19/.27, *p* < .001; Supplementary Table S2).

### Univariate associations

Regarding univariate associations, momentary QoL was not significantly related to session of measurement (*B* = .05, CI − .01/.11, *p* = .123), age (*B* = − .06, CI − .12/.1, *p* = .081), gender (*B* = .01, CI − .05/.07, *p* = .72), Barthel Index (*B* = − .006, CI − .07/.06, *p* = .86), and FAST score (*B* = .04, CI − .03/.10, *p* < .240; Table 2). However, we did find a significant positive association of momentary QoL with comprehensive QoL (*B* = .17, CI .11/.23, *p* < .001) and a significant negative association of momentary QoL with GDS (*B* = − .16, CI − .22/− .10, *p* < .001).

Regarding the random effects in the univariate model, where momentary QoL was regressed on comprehensive QoL, the random variance of session of measurements between PwD (autoregression, diagonal) was significant in the univariate model (*B* = .90, CI .84/.96, *p* < .001), as well in the multiple model (*B* = .90, CI .82/.95, *p* < .001; Table 2). Furthermore, the residual correlation between two sessions of measurement was significant in the univariate model (autoregression, rho; *B* = .39, CI .35/.43, *p* < .001), as well as in the multiple model (*B* = .37, CI .33/.41, *p* < .001), indicating that a higher-than-average rating in one session is associated with a higher-than-average rating on the consecutive session. However, the random variance between facilities was neither significant in the univariate model (*B* = .08, CI .03/.22, *p* = .062), nor in the multiple model (*B* = .09 CI .03/.25, *p* = .058).

### Multiple associations

For the multiple associations of momentary QoL with the comprehensive QoL, momentary QoL was not significantly related to session of measurement (*B* = .03, CI − .02/.09, *p* < .264); Barthel Index (*B* = − .02, CI − .11/.06, *p* < .602); FAST (*B* = .04, CI − .04/.13, *p* < .358); age (*B* = − .06, CI − .13/.00, *p* = .054); and gender (*B* = .03 CI − .03/.09, *p* < .321). However, the momentary QoL was significantly positively related to comprehensive measured QoL at baseline (*B* = .14, CI .08/.20, *p* < .001) and significantly negatively related to GDS (*B* = − .13, CI − .19/− .06, *p* < .001).

Finally, concerning associations of QUALIDEM subscales with comprehensive QoL, positive (*B* = .17, CI .11/.23) and negative affect (*B* = .13, CI .07/.19), restlessness (*B* = .07, CI .01/.14) ,as well as social relationships (*B* = .16, CI .09/.22) and isolation (*B* = .07, CI .01/.14) were significantly related (all p > .05; see Table 3).

## Discussion

Validating a short form of QUALIDEM for purposes of in the moment assessment of QoL and changes in QoL over time, we hypothesized a positive relation between momentary and comprehensive QoL with and without adjusting for covariates. While internal consistency of the momentary QoL was demonstrated, we found univariate and multiple associations of momentary and comprehensive QoL suggesting QoL can be assessed validly. In the multiple case, momentary QoL was significantly negatively related with GDS. Multiple associations of momentary QoL with comprehensive QUALIDEM subscales indicated positive and negative affect and restlessness, and social relationships and social isolation were significantly positively associated with momentary QoL, whereas the others were not.

In our multiple analysis, we found that GDS was substantially and negatively related with momentary QoL. This finding was in line with majority of the research that investigated mood, depressive symptoms, affective status, and happiness [39–41]. Further, most studies showed comparable results investigating multidimensional associations of the QUALIDEM subscales [25–30]. Moreover, in our study, we did not find significant outcomes regarding gender, age, FAST, and Barthel Index, which contradicts findings from the literature [42, 43]. Thus, more research is needed to examine the association of momentary QoL and other indicators of health and functioning.

Furthermore, we found subscales for positive and negative affect, restlessness, and social relationships that were significantly related with momentary QoL. This is largely in line with findings from the MEDLO study, showing that being in a positive mood and engaging in social interactions at state-level assessment was related to a higher QoL assessed on an individual level in PwD in nursing homes [13, 44]. Further studies showed that social engagement is essential for PwD and related with increased levels of QoL [40, 45]. Beerens et al. (2018) suggested focusing on the type of social interactions and rating the quality of interaction [44]. The subscales positive self-image, care relationship, and having something to do were not significantly associated with momentary and comprehensive QoL. This is in line with the literature because in people with very severe dementia the domains such as positive self-image, having something to do, and feeling at home cannot be assessed [46].

Nordheim et al. (2015) showed in their pilot study that the use of tablet computers by PwD living in nursing homes improved the contact to family members and the nursing staff. Additionally, the well-being of residents had been improved [47]. Additionally, technology may be assistive for PwD and can also be used as a telecare service in a home care setting. Furthermore, a combination of several technologies should be investigated regarding momentary and comprehensive QoL and other covariates (GDS, Barthel Index and FAST) of PwD living in nursing homes [48].

Dementia-specific assessment of QoL is multidimensional and depends on the individual environment of PwD. Adaption influences the rating of QoL of PwD living in nursing homes and are described in the adaptive coping model [31]. Ettema et al. (2007) described the life domains of QUALIDEM that were chosen by consensus [41]. Lawton et al. (1991) considered the well-being of elderly people as the main outcome which is affected by the person-environment system of PwD [49]. Due to this importance of variables that affect QoL, we took different variables into account. Momentary and comprehensive QoL were examined. Nursing home of every PwD had been used as a clustering variable to detect effects between the ten nursing homes. QoL instruments such as QUALIDEM had not yet been tested completely to investigate psychometrical variables.

## Strengths and limitations

Strengths of our study include the ecological design with a large number of observations across time and an innovative and relevant research topic, which may inform future QoL assessment in research and practice. In our study, limitations include the moderate number of long-term care facilities that may have covered the potential between-facility effects, which should be tested in future studies. Additionally, we were not able to measure other scales such as FAST or Barthel Index at momentary level; thus, time-lagged associations across scales at momentary level could not be detected in the present study design. Another limitation concerns the exclusion of important aspects of QoL, such as social isolation. The subscale ‘social isolation’ was represented in our sum score of the 37-item version of QUALIDEM with the item 16: ‘Is rejected by other residents’, item 20: ‘Openly rejects contact with others’, and item 32: ‘Calls out’. Previous studies of reliability and validity between the 18-item version and the 37-item version of QUALIDEM showed that the subscales ‘having something to do’ and ‘social isolation’ were weakly or not scalable, according to the Loevingers coefficient of homogeneity and scalability [9, 38]. However, the subscale ‘social isolation’ was associated with our momentary QoL scale; thus, the short version may at least cover some aspects of social isolation. This might be due to the fact that communication, sociability, and sadness, which likely cover some aspects of social isolation and loneliness, were included in our short version. Nonetheless, future studies should inspect the further role of ‘social isolation’ and ‘social relationships’ in the eight-item version of QUALIDEM.

## Implications for research and practice

Mobile technologies may help to monitor patients with mild and severe dementia or other clinical situations and can be a low-cost option to support caregivers of PwD in a non-clinical setting. Although intervention apps for PwD may be time intensive, it might save time, if the technology would be established and integrated in the activities of daily life [50]. Our eight-item Version QUALIDEM which represents the domains of QoL had been examined as a reliable and valid tool to assess QoL in people with mild and severe dementia living in nursing homes. It may be used by clinical staff in regular bases to assess and diagnose residents of nursing homes every day [51].

Nevertheless, more longitudinal studies are needed to determine if more factors are related to a change in QoL over time. This information could be important for the development of interventions that aim to improve QoL and for diagnosing and daily assessment of QoL of PwD living in nursing homes [13]. Other tools for the assessment of QoL may be compared and evaluated with our method as well. Furthermore, more touchscreen interventions should be conducted to compare those with our method and evaluate QoL including variables such as the QUALIDEM subscales. Moreover, studies using technology-based assessment tools outline the advantages such as accuracy, efficiency, acceptability, and feasibility [18–21].

A positive association of momentary QoL and comprehensive QoL indicates that a touchscreen-based assessment instrument could be used to measure mood and social engagement of PwD [15].

According to other subscales such as positive self-image and care relationship, we found no association with momentary QoL, which is likely to be related to the selection of the eight state items from subscales that were not related with self-image and care relationship. However, both subscales were unlikely to vary substantially across short periods of time, which may be a further explanation for the non-significant relationship. Nonetheless, future studies should inspect the relationship between perceived care relationship and momentary QoL, being uncovered by our findings. Studies demonstrated that mood of PwD was correlated with factors such as unfulfilled needs or environmental factors [13]. Thus, investigating activity categories in future studies is needed.

The negative association of GDS and momentary QoL is important, as depression is a common comorbid disorder of dementia affecting between 23 and 54% of PwD, which is substantially higher compared to the general population [52, 53]. Thus further studies are necessary to find more relationships between the use of technology-based interventions and depression in PwD in nursing homes [17].

Robertson et al. showed that staff who were more distressed rated QoL of PwD lower than those raters being less distressed [54]. Further studies are necessary to find effects of raters on the variables that we are targeting for. Broader use of tablet-based assessments may improve the time management of nursing staff. This conclusion is in line with the study by Muller et al. that considered tablet-based assessment of dementia and mild cognitive disorders as an efficient assessment tool to diagnose dementia faster [55]. Using the short eight-item version of QUALIDEM for momentary assessment of QoL in our study showed good reliability; therefore, we suggest the broader implementation of the short eight-item version of QUALIDEM in further studies or clinical settings. The gain of data may improve the care quality and communication between staff and PwD. Future studies may investigate the use of tablet-based interventions in nursing home environments or other clinical settings, to evaluate QoL not only in PwD, but in other geriatric patient groups as well. In that case, disease-specific instruments should be applied [19, 51, 56, 57].

## Conclusions

We found that momentary QoL was associated with comprehensive QoL as well as depressive symptoms in PwD living in nursing homes. The use of tablet-based assessments, especially the short eight-item version of QUALIDEM may enhance our knowledge on the mechanisms of QoL over time and may improve assessment by nursing staff and ultimately QoL in PwD.

## Electronic supplementary material

Below is the link to the electronic supplementary material.

Supplementary file1 (DOCX 15 kb)
