# Supplementary material for: Quality of life in people with dementia living in nursing homes: validation of an eight-item version of the QUALIDEM for intensive longitudinal assessment
Source: Qual Life Res. 2020 Jan 18;29(6):1721–30. doi: 10.1007/s11136-020-02418-4 (PMC7253522; doi:10.1007/s11136-020-02418-4)
Supplement: Supplementary file 1 — Supplementary file1 (DOCX 15 kb) [file 11136_2020_2418_MOESM1_ESM.docx]

**Quality of life in people with dementia living in nursing homes: Validation of an eight-item version of the QUALIDEM for intensive longitudinal assessment**

Stefan Junge^1(^0000-0003-3150-1505), Paul Gellert^1^(0000-0001-7492-7210), Julie Lorraine O'Sullivan^1^, Sebastian Möller^2^, Jan-Niklas Voigt-Antons^2^(0000-0002-2786-9262), Adelheid Kuhlmey^1^ (0000-0003-4088-4675), Johanna Nordheim^1^

Corresponding author email: stefan.junge@charite.de ; Tel.: +49 30 450 529 215

**Supplementary TABLES**

| **Supplementary Table S1. Internal consistency of state-level quality of life by time point** | | | | | | | | | | | | | | | |
| --- | --- | --- | --- | --- | --- | --- | --- | --- | --- | --- | --- | --- | --- | --- | --- |
| *Time point* | 0 | 1 | 2 | 3 | 4 | 5 | 6 | 7 | 8 | 9 | 10 | 11 | 12 | 13 | 14 |
| *Cronbach’salpha* | .89 | .88 | .88 | .88 | .92 | .90 | .91 | .93 | .93 | .92 | .94 | .92 | .89 | .90 | .92 |
|  |  |  |  |  |  |  |  |  |  |  |  |  |  |  |  |
| *Time point* | 15 | 16 | 17 | 18 | 19 | 20 | 21 | 22 | 23 | 24 | 25 | 26 | 27 | 28 |  |
| *Cronbach’salpha* | .90 | .91 | .94 | .90 | .95 | .96 | .93 | .97 | .90 | .98 | .99 | .88 | X | X |  |

| **Supplementary Table S2. Retest-reliability of state-level quality of life** | | | | |
| --- | --- | --- | --- | --- |
|  | Beta | P-value | CI min | CI max |
| *Model 1* |  |  |  |  |
| State-level QoL, lag1 | .40 | <.001 | .36 | .44 |
| *Model 2* |  |  |  |  |
| State-level QoL, lag2 | .36 | <.001 | 31 | 40 |
| *Model 3* |  |  |  |  |
| State-level QoL, lag1 | .32 | <.001 | .27 | .36 |
| State-level QoL, lag2 | .23 | <.001 | .19 | .27 |
| *Note.* Lag1=association of time point (t) with one time point lagged (t-1); Lag2=lagged across two time points (t-2) | | | | |
